# Error

**Published:** 1882-11-20

**Authors:** 


### EDITORIALS AND MISCELLANEOUS.

ERROR.—In our August number, page 293, there is a mistake—copied from an exchange—which every subscriber should turn to and correct. It should read—

℞ Bismuth subnit.......................................... ʒ ijss,

Pulv. creta, aromat. cum. opii.......................... ʒ ss,

Pepsin sacch.......................................... ʒ ij.

M. Make 8 powders. As it originally stands the opium is in dangerous proportion.

